# Field Metabolic Rate and PCB Adipose Tissue Deposition Efficiency in East Greenland Polar Bears Derived from Contaminant Monitoring Data

**DOI:** 10.1371/journal.pone.0104037

**Published:** 2014-08-07

**Authors:** Viola Pavlova, Jacob Nabe-Nielsen, Rune Dietz, Jens-Christian Svenning, Katrin Vorkamp, Frank Farsø Rigét, Christian Sonne, Robert J. Letcher, Volker Grimm

**Affiliations:** 1 Aarhus University, Department of Bioscience, Section for Marine Mammal Research, Roskilde, Denmark; 2 Aarhus University, Arctic Research Centre, Aarhus, Denmark; 3 Aarhus University, Department of Bioscience, Section for Ecoinformatics and Biodiversity, Aarhus, Denmark; 4 Aarhus University, Department of Environmental Science, Section for Environmental Chemistry and Toxicology, Roskilde, Denmark; 5 Helmholtz Centre for Environmental Research - UFZ, Department of Ecological Modelling, Leipzig, Germany; 6 University of Potsdam, Institute for Biochemistry and Biology, Potsdam, Germany; 7 Ecotoxicology and Wildlife Division, Science and Technology Branch, Environment Canada, National Wildlife Research Centre, Carleton University, Ottawa, Ontario, Canada; Northwest Fisheries Science Center, NOAA Fisheries, United States of America

## Abstract

Climate change will increasingly affect the natural habitat and diet of polar bears (*Ursus maritimus*). Understanding the energetic needs of polar bears is therefore important. We developed a theoretical method for estimating polar bear food consumption based on using the highly recalcitrant polychlorinated biphenyl (PCB) congener, 2,2′,4,4′,55-hexaCB (CB153) in bear adipose tissue as an indicator of food intake. By comparing the CB153 tissue concentrations in wild polar bears with estimates from a purposely designed individual-based model, we identified the possible combinations of field metabolic rates (FMR) and CB153 deposition efficiencies in East Greenland polar bears. Our simulations indicate that if 30% of the CB153 consumed by polar bear individuals were deposited into their adipose tissue, the corresponding FMR would be only two times the basal metabolic rate. In contrast, if the modelled CB153 deposition efficiency were 10%, adult polar bears would require six times more energy than that needed to cover basal metabolism. This is considerably higher than what has been assumed for polar bears in previous studies though it is similar to FMRs found in other marine mammals. An implication of this result is that even relatively small reductions in future feeding opportunities could impact the survival of East Greenland polar bears.

## Introduction

Polar bears *(Ursus maritimus)* are currently listed as vulnerable on the International Union for Conservation of Nature and Natural Resources (IUCN) red list, with the main threats identified as global climate change, pollutants, oil exploration, and hunting [1]. As an apex predator that predominantly feeds on ice dwelling seals, the polar bear is highly dependent on the presence of sea ice, which forms their platform for hunting [2]. In the near future, the potentially longer sea-ice free periods in the Arctic present a risk of food shortage that could directly influence the viability of bear subpopulations [3], [4]. The health of bears, litter mass, litter size, survival, and overall subpopulation size could be affected [5], [6], [7], [8], [9]. For effective conservation it is therefore essential to understand polar bear feeding habits and energetic needs. The diet composition of polar bears has already been studied extensively. Polar bears predominately eat the blubber and meat of ringed seals *(Phoca hispida)* or other seal species like the bearded (*Erighnatus barbatus*), harp (*Pagophilus groenlandica*) and hooded seal (*Cystophora cristata*) as well as other marine mammals [2], [10], [11], [12], [13], [14], [15].

While it is important to know the composition of the polar bear diet, it is also crucial to know their overall energy needs. The energetic requirement is dependent on the field metabolic rate (FMR) that polar bears exhibit. The FMR accounts for basal metabolism and any additional energy requirements in connection to movement and other activities in their natural habitat. Measuring FMR in free-ranging animals is possible using the doubly tritium labelled water method or heart rate measurements [16], [17], however, to our knowledge, such measurements have not been done for wild polar bears. Studies undertaken during the summer in the high Arctic reported the field metabolic rate of polar bears to range between 2.0–2.6 times the basal metabolic rate (BMR) [18], [19], or between “12 to 16,000 kcal/day” using captive bear feeding trials. This represents 50,400 to 67,200 kJ/day. Further estimates of biomass consumption have been inferred by extrapolating from ringed seal population data and from polar bear behaviour studies [10], [20], [21]. Metabolic costs related to locomotion have also been investigated in captive polar bears and it has been shown that movement increases the metabolic rates considerably [22], [23]. It remains unclear how these estimates reflect the annual cycle of wild polar bears.

To our knowledge, no study on polar bear energetics has been undertaken in East Greenland and it is thus unknown how closely the estimates of energetic demands from other areas or captive bears would match the situation there. Our study was designed to provide independent insight into polar bear energetics using a new approach and with a focus on the subpopulation in East Greenland. We developed a theoretical method that is based upon using the concentration of an accumulated and recalcitrant contaminant (polychlorinated biphenyl (PCB) congener, 2,2′,4,4′,5,5′-hexaCB (CB153)) in polar bear adipose tissue as a chemical marker of polar bear food intake rate. Many studies have documented high levels of PCBs in polar bear tissues in the East Greenland subpopulation [24], [25], [26], [27], [28]. The PCBs are predominantly acquired through the diet [29], [30]. Over the past decades the PCB concentrations in the East Greenland ringed seals, the dominant food source of polar bears [15], have been decreasing [31], [32]. The concentrations in adipose tissues of the polar bears in East Greenland follow the trend accordingly [27]. The diet composition may influence the contaminant burden of the bears, but most types of food are likely to contain some level of PCBs [28], [33]. The final adipose tissue residues of the highly recalcitrant PCB congeners depend on the amount of food consumed and its contamination level, as well as on the efficiency of deposition of the contaminant into the adipose tissues (hereafter referred to as deposition efficiency), which is an outcome of internal physiological pharmacokinetic processes [34]. In females the total body burdens are further affected by the lactation transfer of contaminant to the offspring [35]. The PCB deposition efficiency has not been measured in polar bears, but the closely related grizzly bears (*Ursus arctos horribilis*) deposit only about 10% of the ingested PCBs [36] while the rest is bio-transformed or excreted. Given the relatedness of grizzly and polar bears the PCB deposition efficiency could be similar in the two species, but no conclusions could be made based on available literature.

The objective of the study was therefore to estimate the possible FMR in relationship to the deposition efficiency of CB153 in East Greenland polar bears. The basic idea of our approach was to infer possible combinations of field metabolic rates and deposition efficiency based on data on CB153 in ringed seal blubber taking into account lactation contaminant transfer.

## Methods

The present study had the following overall methodology. First, in order to obtain the input for our model, we analysed time series data on CB153 concentration in blubber of East Greenland seals and calculated their growth parameters (section 2.1 *Ringed seal contamination & growth*). Second, we implemented a simple individual-based model representing bioaccumulation of polar bears preying on seals. The model is not a physiologically based pharmacokinetic model, but accounts for direct transfer from prey to bears, and to offspring through lactation. The model predicted the CB153 adipose tissue concentrations between 1986 and 2009 (section 2.2 *Model description*). With this model we ran numerous simulations with varying combinations of food intake level and CB153 deposition efficiency. We then compared the model predictions of CB153 concentration in the population over multiple years to actual field data on CB153 concentrations in East Greenland polar bears to determine which of the parameter combinations produced CB153 loads similar to those observed over time in East Greenland polar bears (section 2.3 *Analysis of model predictions*). A test of model sensitivity was also performed (section 2.4 *Local sensitivity analysis*). For all statistical analyses in this study we used the R language, version 3.0.0 (R Core Team 2012). The values and references to all model parameters are presented in *Table S1. Parameter values and references* in Supporting Information (SI).

### 2.1 Ringed seal contamination & growth

The field data on CB153 concentrations in blubber of ringed seals in East Greenland were collected in the years 1986, 1994 and every year in the period 1999–2004 and in 2006, 2008 and 2010 (collection methods are described in [31], [32], [37]. We used this data set to predict the age- and time-dependent concentrations of CB153 for every year and each age class of seals during the period between 1986 and 2009. The data consisted of information on sex, age, weight, length, blubber depth, and concentration of CB153 of each sampled seal. We divided the seals into 12 age classes: 0, 1…10 years old, and 11+ for seals 11 years old and older, because there were only few individuals of older age. The log-transformed CB153 concentrations in seal blubber and seal age classes were then fitted with a linear model: log_10_ [CB153] ∼ year * age class (F12,232 = 7.518, p<0.0001. R^2^ = 0.28). The concentrations predicted by this model decreased in all age classes throughout the study period (details available in Supporting Information (SI): *Figure S1* and *SI Text S1. Ringed seal contamination & growth*). The same dataset was also used to find the age – weight growth parameters of seals in East Greenland assuming a von Bertalanffy growth curve (Equation [S1], *SI Text S1, values of parameters shown in Table S1*), which has previously been used for ringed seals in Svalbard [38], [39]. We also estimated the amount of blubber present on each seal of certain age class (*Text S1*). The lists of the model predicted CB153 concentrations for seal blubber are available within the model code (*available as a text file (File S1) or as a NetLogo file (File S2)*).

### 2.2 Model description

A detailed model description, following the ODD (Overview, Design concepts, Details) protocol for describing individual-based and agent-based models [40], [41] is provided in *SI Text S2. Overview, Design & Details (ODD) protocol*. The main purpose of the model was to produce predictions of CB153 concentrations in adipose tissue of a whole subpopulation of polar bears in East Greenland between 1986 and 2009. The model did not account for any competition for food or any behavioural or other interactions among polar bears (such as territoriality, mating etc.), except for the transfer of contaminants between female bears and their offspring. For implementation we used the Net Logo 4.1 platform [42]; the code is available in the Supporting Information (*File S1, File S2*).

The model simulated polar bear individuals from 2 to 30 years of age. Bears were characterized by their age, sex, yearly energy requirement, weight, the proportion of storage blubber and the total body burden of CB153. Polar bear females and males coexisted in the model, but male agents engaged only in selected processes: they grew and fed according to their weight, accumulated the contaminant and died. Because females produced offspring their energy needs and contamination burdens were influenced by fluctuation in weight related to reproduction, by milk production and resulting lactation transfer. Females therefore had additional variables representing pregnancy status (i.e. pregnant or not), reproductive status (single, with cubs, with yearlings) and the number of offspring in current litter. The offspring were either cubs or yearlings dependent on the reproductive status of their mother. Information on the CB153 body burdens of the offspring, energy requirements per yearling per annum, and on the number of days of survival for each offspring was also stored as a variable of each female. Cubs and yearlings were not represented as individual agents because their contamination levels were dependent on the contamination levels of their mothers. The model did not incorporate any spatial movement of individuals or any variation in food or bear properties that would result from their spatial distribution. The time step was one year. We simulated the period between 1986 and 2009. The only environmental variable was the content of CB153 in ringed seals.

Below we describe processes simulated in the model as well as some algorithms and equations essential for the understanding of the main drivers determining CB153 contamination in the model bears. All further equations and their rationale are included in the ODD protocol in the Supporting Information (*Text S2*). For parameter values and references, see *Table S1*. During each time step, the following processes were simulated in the given order:

Firstly, offspring survival and pregnancy of mature females during the current year was determined according to survival and breeding probabilities originating from [43] (*Table S1*). (Submodel ***Offspring survival and pregnancy***, *SI Text S2*).

Secondly, weight and blubber content of each individual was updated according to sex, age and reproductive status. The bear weight was calculated according to the von Bertalanffy growth function with parameters for polar bears following [44] (*Table S1*). (Submodels ***Update weight*** *&* ***Update blubber*** *, SI Text S2*).

Third, the energy requirements of each bear were calculated according to its reproductive status, weight and, in female bears, number of days of lactation. The annual energy requirement of older bears (older than a yearling), *E_A_* [kJ], was assumed to equal to 365 times the field metabolic rate [kJ/day]. The latter was calculated using Kleiber's rule [45] for basal metabolic rate [kJ/day] multiplied by a factor *f_A_* that accounted for excess energy needed for movement and activity:
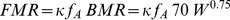
(1)where *W* is the mass [kg] of the bear and κ was set to 4.2 and converts kilocalories to kilojoules. The factor *f_A_* is henceforth referred to as the ‘*field metabolic factor*’. We thus assumed that older bears required the following amount of energy per annum [kJ]:
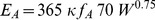
(2)where *W* is the mass [kg] of the bear and *f_A_* is the field metabolic factor bear and κ was set to 4.2 and converted kilocalories to kilojoules. Ten different values [1], [2]–[10] of the field metabolic factor (chosen with respect to values published for other marine mammals [17], [53], [54]) were tested. The annual energy requirement of cubs was assumed to be equal to the amount of energy contained in mother's milk produced during one year (*Text S2, p. 6–7*), while for yearlings the above presented formula was applied, but using a separate field metabolic factor *f_Y_*. In this case, after running initial trials we decided to test a slightly wider span of values [1], [2]–[13]. The energy requirements of yearlings were partly covered by nursing and partly by eating the seal blubber. Further on, the yearly energy demands of females with cubs or with yearlings were equal to *E_A_* plus the total amount of energy contained in the milk produced by the female during the year, which depended on the number and age of their offspring (*Text S2, eq. S5, S6*). The above described calculation was solely for the purpose of determining the amount of food and hence the amount of contaminant ingested annually by the bears and was not related to survival or breeding probability. (Submodel ***Set energy requirements***, *Text S2*).

As a next step, each bear satisfied its annual energy requirements by feeding exclusively on ringed seal blubber, which was assumed to consist only of lipids and to have been totally digestible. The blubber was acquired from individual seals caught in an iterative process until the bears satisfied their energy needs. The caught seals were of random sex, but their age was determined according to the documented hunting preferences of polar bears for seal pups, one to two year old seals and three to nineteen year old seals [20] (*Table S1*). With each seal consumed, the bears correspondingly increased their CB153 burden by the amount that was present in the seal blubber multiplied by the deposition efficiency (*A_A_* for all bears two years old and older, hereafter referred to as “older bears”). To explore the whole range of possible values of deposition efficiency we tested ten values [0.1, 0.2–1] representing the storage of [10, 20, -100]% of ingested contaminant into adipose tissue. We assumed that all bears satisfied their energy needs and that there was no competition for food (Submodel ***Feed***, *SI Text S2*).

The CB153 body burdens of lactating females were reduced by the amount of contaminant transferred to the milk (*Text S2, eq. S12, S14*). This amount was partially retained by the offspring, depending on the deposition efficiency *A_C_* for cubs and *A_Y_* for yearlings. Because the A_c_ value was unknown and could not be determined via calibration, we used estimations published by [46] on the amount of sum PCBs ingested by cubs and the resulting residues in their tissues, and estimated the value to be 0.23 (*Table S1*). In yearlings we tested values: [0.1, 0.2–1], It should be noted that because no relevant data was available we neglected any trans-placental transfer of the contaminant in pregnant females. (Submodel ***Lactation transfer***, *SI Text S2*).

Finally, population dynamics were addressed as follows: all individuals >30 years died, in addition to a random selection of the remaining bears (according to survival probabilities from [43], *Table S1*). New individuals were created for weaned yearlings that were approaching two years of age. The reproductive status of females was updated (Submodel ***Population dynamics***, *SI Text S2*).

As the last step age was updated as well as was weight and proportion of storage blubber (Submodels ***Update age*** *,* ***Update weight, Update blubber***, *SI Text S2, Table S1*).

To initialize simulations, we started with 2000 individuals with random age between 2 and 20 years and randomly assigned sex and in females the initial pregnancy status (pregnant or not) and the number of offspring. The initial body burdens of all bears were set to zero and therefore (and in order to reach a stable age structure) the simulations were started already in 1950. This allowed contamination loads to build up to the 1986 level. Only the output for the period between 1986 and 2009 was used because no data were available for either seals or bears for the previous period. The contamination load of seals from 1986 was used as an approximation for the period between 1950 and 1986 as input due to lack of monitored data from this period.

### 2.3 Analysis of the model output

#### Time trend analysis

We investigated the influence of various combinations of the field metabolic factor (for both yearlings and older bears) and of the deposition efficiencies of the two groups on the individual adipose tissue concentrations of CB153 in the bear subpopulation. The best parameter combinations were sought by comparing the linear regression lines of a random subsample of individual CB153 concentrations for periods between 1986 and 2009 with the real observations from East Greenland polar bears collected during the same time period (with gaps). The methods of data collection and a thorough time trend analysis of the collected data were provided by Dietz et al. [27]. For the purpose of our analysis, the original data was subsampled to 13 yearlings and 295 individuals two years old and older; one outlier with extremely low CB153 concentration was excluded due to suspected measurement errors. Although the maximum age of bears in the model was 30 years, we included only individuals younger than 26 years in the analysis from both the empirical data and model predictions. This was necessary, because the oldest bears formed a very small proportion of the East Greenland sample compared to model output. Cubs also had to be excluded from the analysis, because there were too few in the empirical data. Data for yearlings were not available for the whole period and were thus compared only for the period between 1987 and 2000. Yearlings and older bears formed two separate groups in this analysis.

We tested all combinations of *f_A_* (values 1, 2 … 10), *f_Y_* (values 1,2,…13) and *A_A_* and *A_Y_* (both 0.1, 0.2… 1) (total number of simulations: 13,000). Each simulation (*s*) represented a unique combination of the four parameters. The individual CB153 concentrations were log transformed. To find the combinations of parameters producing time series data with regression lines best matching those of the data collected in Greenland for both the yearlings and the older bears, we used ANCOVA and tested the model **ln (** ***CB153 concentration*** ) ∼ year * simulation for differences in slope and intercept. The predictor variable (year) was centred in the linear regression so that the intercept could be interpreted as the value of the dependent variable for the centre of the time interval, rather than for year 0 (which would not be useful). We tested the model separately for yearlings and older bears, and only if the results were not significantly different for both categories (using significance levels *p*>0.1) was the simulation accepted as producing contaminant levels similar to those observed in real polar bears. The parameter combinations that produced these results were further assessed for biological relevance in order to identify the most probable parameter combinations.

#### Model validity

In order to verify that the matching time-trend predictions as identified in previous steps were not an artefact of data pooling, we divided the bears into subgroups according to their age category and sex: yearling, subadult females, subadult males, adult females and adult males. Then we compared the medians of predicted CB153 concentrations within these subgroups with the East Greenland data. This allowed us to check whether the results produced by the model also corresponded with the real observations with respect to the sex and age of bears. The identification of independent and realistic properties emerging from the model was used to support the validity of principles and assumptions used in our model [47].

### 2.4 Local sensitivity analysis

We analyzed the influence of changing each parameter in the model by ±10% relative to its reference value (f_A_ = 6, f_Y_ = 11, A_A_ = 0.1, A_Y_ = 0.3 and other parameters as in Table S1) on the mean body burden of yearlings and older bears. Only in case of the survival probability σ_y_ a change of ±9% had to be used as the original value was too close to 1. The resulting change (in%) was determined for the year 1999. Five simulations were run for each parameter combination and their results averaged.

## Results and Discussion

**Figure 1:**
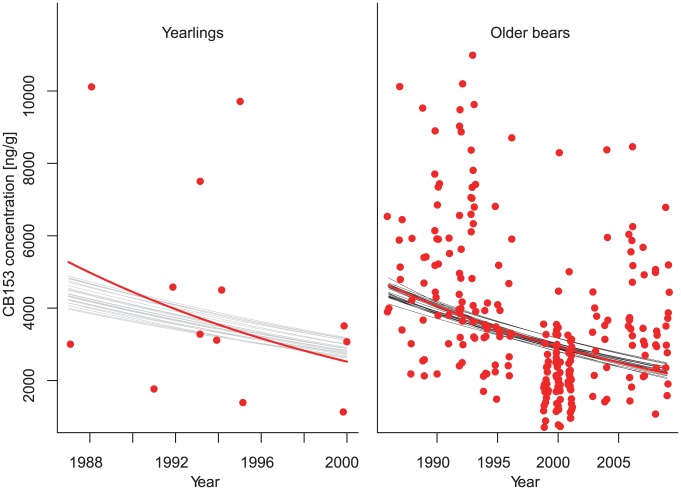
Time trend in polar bear CB153 concentrations. Red dots show the CB153 concentrations in individual East Greenland bears across the study period. Red curve shows log – linear trend in the data. Grey and black curves show simulated trends in CB153 concentrations produced using parameter combinations that produced predictions that did not differ from the observed trends in East Greenland for yearlings and older bears, respectively.

Only 24 of the 13,000 tested combinations of field metabolic factor *f_A_* and *f_Y_* and deposition efficiencies *A_A_* and *A_Y_* produced CB153 concentrations in the bear population that were not significantly different from the East Greenland observations (Table 1). The CB153 concentrations in East Greenland polar bears decreased during the study period and the 24 parameter sets described above reproduced this pattern accordingly (Fig. 1).

**Table 1:** ANCOVA selected parameter combinations.

Nr.	*A_Y_*	*A_A_*	*f_Y_*	*f_A_*
*1*	*0.3*	*0.7*	*8*	*1*
*2*	*0.3*	*0.7*	*9*	*1*
**3**	**0.3**	**0.3**	**9**	**2**
**4**	**0.3**	**0.3**	**10**	**2**
**5**	**0.3**	**0.2**	**10**	**3**
**6**	**0.3**	**0.1**	**10**	**6**
**7**	**0.3**	**0.1**	**10**	**7**
*8*	*0.4*	*0.6*	*7*	*1*
*9*	*0.4*	*0.7*	*6*	*1*
**10**	**0.4**	**0.3**	**7**	**2**
*11*	*0.5*	*0.6*	*5*	*1*
*12*	*0.5*	*0.7*	*5*	*1*
*13*	*0.6*	*0.6*	*5*	*1*
**14**	**0.6**	**0.3**	**5**	**2**
*15*	*0.6*	*0.1*	*4*	*7*
*16*	*0.7*	*0.6*	*4*	*1*
*17*	*0.7*	*0.8*	*3*	*1*
*18*	*0.8*	*0.7*	*3*	*1*
*19*	*1*	*0.1*	*2*	*7*
*20*	*0.3*	*0.6*	*11*	*1*
**21**	**0.3**	**0.3**	**11**	**2**
**22**	**0.3**	**0.1**	**11**	**6**
*23*	*0.2*	*0.7*	*12*	*1*
*24*	*0.2*	*0.7*	*13*	*1*

Parameter combinations that produced CB153 contamination patterns not significantly different from those of East Greenland polar bears (biologically relevant combinations where *f_A_*>1 or *f_Y_*≥*f_A_* are in bold font, non-relevant combinations in italics).

**Figure 2:**
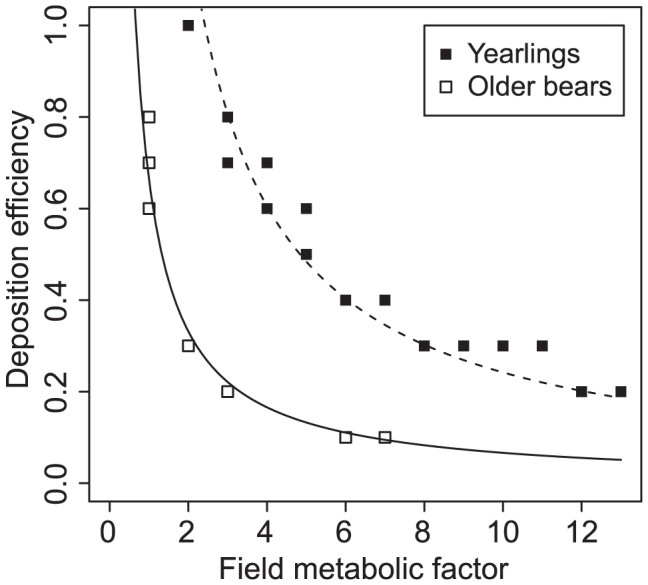
Relationship between deposition efficiency and field metabolic factor. Within the parameter combinations that produced matching predictions the deposition efficiency was inversely proportional to the filed metabolic factor in both groups; the yearlings and older bears. Solid curve: *A_A_ = 0.6626/f_A_*, dashed curve: *A_Y_ = 2.42/f_Y_*.

Among the ANCOVA selected parameter combinations (Table 1) the deposition efficiencies in older bears and yearlings were inversely proportional to field metabolic factors:

*A_A_ = ω_A_/f_A_* and *A_Y_ = ω_Y_/f_Y_*, where *ω_A_* and *ω_Y_* are the proportionality constants (equal to the products of the studied parameters for adults: *ω_A_* = *A_A_*×*f_A_* and yearlings: *ω_Y_ = A_Y_*×*f_Y_*) with values of *0.6626* and *2.42* respectively (Figure 2).

**Figure 3:**
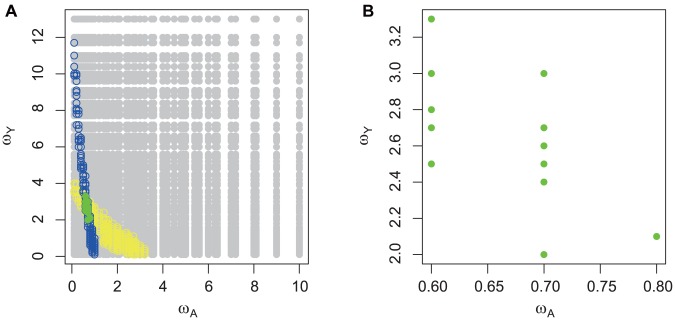
Tested combinations of deposition efficiencies and field metabolic factors. a) Only few combinations of proportionality constants: *ω_A_* ( = *A_A_*×*f_A_*) and *ω_Y_*(* = A_Y_*×*f_Y_*) produced predictions not significantly different from actual data: in older bears (blue), yearlings (yellow) or both groups (green). All tested combinations are coloured grey. b) Combinations of *ω_A_* and *ω_Y_* that produced predictions not significantly different from actual data in both studied groups exhibited a negative relationship.

Visual representation of all values of proportionality constants *ω_A_* and *ω_Y_* allowed us to gain a better insight into the influence of the combined age group specific parameters on the significance (or non – significance) of the results (Fig. 3a). The contamination levels in yearlings never prevented a realistic contamination time trend to arise for adult bears: with almost any value of *ω_Y_* non-significantly different regression lines in both slope and intercept could be obtained for adults. On the other hand, for yearlings neither *ω_A_* nor *ω_Y_* could be too high. This means that the contaminant levels in older bears determined the contaminant levels in yearlings to a large degree, but not vice versa. The reason is that the body burdens in yearlings formed only a small part of the total body burden of the older bears.

Most importantly, there existed only narrow ranges of *ω_A_* and *ω_Y_* values that resulted in regression lines that were not significantly different from the data for both yearlings and older bears: from 0.6 to 0.8 and from 2 to 3.2, respectively. Within these narrow intervals, *ω_A_* and *ω_Y_* exhibited a negative relationship (Fig 3b), which means that higher levels of contamination in yearlings corresponded to lower levels of contamination in adults.

According to the presented results the deposition efficiency in yearlings or their field metabolic factor (or both at the same time) must be considerably higher than that of adults. Thus, even if the yearlings retained 100% of the contaminants they acquired from food and milk (*A_Y_* = 1) their field metabolic rate would still have to be 2.0 to 3.2 higher than their basal metabolic rate. On the other hand if the older bears exhibited 100% deposition efficiency, their field metabolic rate should only be about 66% of their basal metabolic rate. This indicates that there must be a considerable loss of the contaminant between its ingestion and incorporation into the adipose tissue in older bears.

While 24 parameter sets were identified as producing realistically decreasing contaminant loads for the period 1986–2009, some of these are not biologically justifiable, as often is the case for calibrated parameter sets [48]. Thus, all sets with *f_A_* equal one, where the field metabolic rate would equal to the basal metabolic rate, can be dismissed as unlikely as well as the few combinations where the per unit body weight energy requirement of yearlings would be smaller than that of older bears (Table 1, combinations in italics). In the remaining solutions the parameters of older bears typically occur in two combinations *f_A_* = 2 or 3 with *A_A_* = 0.3 or *f_A_* = 6 or 7 with *A_A_* = 0.1, while the field metabolic factor for yearlings, *f_Y_*, ranges between 9 and 11 in most cases, with a corresponding deposition efficiency *A_Y_* of 0.3. In two occasions *f_Y_* is lower, reaching only 5 and 7 and the corresponding deposition efficiency is thus higher: 0.6 or 0.4.

In theory, these results could support the field metabolic rate of 2–2.6 ^x^ BMR as suggested for polar bears previously [18], [19]. The older East Greenland polar bears should then have incorporated about 30% of the ingested CB153 into their adipose tissues. However, if the deposition efficiency were as low as in grizzly bears [36], with the bears retaining only about 10% of the contaminant, the field metabolic rate of older bears would reach values of 6 to 7 times the basal metabolic rate. This finding is particularly interesting, because it is considerably higher than what has previously been reported for polar bears [18], [19], [20]. We have no data to indicate whether polar bears from different geographical areas might differ in their metabolic rates to such an extent. Stirling and Øritsland [20], used a conservative field metabolic factor of 2 in their polar bear study based on the assumption that polar bears are well adapted to the life in the Arctic and their metabolism can be very efficient. Although the bears often hunt by still-hunting [49], which can be potentially energetically quite inexpensive, they are known to migrate large distances [50] and engage in swimming [51]. Swimming in particular may be energy costly as the insulation qualities of the bear fur strongly decrease in water and the body is then primarily insulated by adipose tissue [52]. The maintenance of sufficient insulation is again likely to require a large amount of food. In other marine mammals rather high field metabolic rates have also been found. Common dolphins (*Tursiops truncatus*) can use up to six times more energy than required for their basal metabolism [53], while FMR values between 5 to 7.4^x^ BMR were estimated for killer whales (*Orcinus orca*) [54]. In walrus (*Odobenus rosmarus*) direct measurements have indicated FMR to be up to 5.5 to 6.5^x^ BMR [17]. This finding was discussed by the authors as conflicting with the common practice of using a field metabolic factor of three for pinnipeds, since it may lead to underestimation in calculations of their food intake [55], [56]. Although polar bears are not close relatives of any of these species, they live in a similarly extreme environment. Using the field metabolic factor of 6 (FMR thus being equal to six times BMR), a bear weighing 200 kg would spend roughly 93,800 kJ/day. This is close to the 95,827 kJ/day predicted for bears based on an allometric relationship developed for non-herbivorous large mammals [57]. With respect to this and the arguments above, a field metabolic factor of 6 seems possible for older polar bears.

The PCB deposition efficiency in yearlings was mostly 30% and the field metabolic factor ranged from 9 to 11 (Table 1). The deposition efficiency is thus the same or somewhat higher than in older bears. This seems plausible, considering that in the PCB deposition efficiency can range from 100% in infants [58] to 10% in adults [59]. We do not know the reasons for this phenomenon in polar bears, but in children immaturity of enzymatic systems or of hepatic and renal functions can cause longer half-lives of xenobiotic [60]. When designing the study, keeping in mind the proposed field metabolic rate of 2^x^BMR from previous studies, we restricted the range of tested field metabolic factor values to 1 to 13, which we assumed covered what seems biologically possible. If we had tested even higher values, we could have possibly found some parameter combination with deposition efficiency as low as 0.1. Nevertheless, according to the current results, juvenile polar bears seem to have an even higher field metabolic rate than older bears.

In dogs (*Canis familiaris*), growing puppies need about 1.8 times more energy per unit body weight compared to adults until they reach 50% of adult weight, and their energy requirement continues to be higher than that of adults until reaching the full body size or even longer [61]. According to published polar bear nutrition guidelines a captive 3-year old polar bear was fed 2 to 3 times more food in relation to its body size than a nine years old bear [62]. Some of the combinations of the parameters (parameter sets 6, 7, 14 and 22 in Table 1) do comply with these ratios. It should be noted though, that over-estimation of the blubber consumption could have occurred due to underestimation of milk consumption rates in cubs and yearlings. It is known that milk production/consumption varies during the year in grizzly bears [63]. Because of lack of data we assumed the daily milk consumption during the whole year to equal to that measured during the summer ice-free period [64]. Furthermore, we have no information on whether the contaminant is absorbed from the milk at a similar rate as from the seal blubber. In the case of cubs totally dependent on milk, we were unable to use our data to calibrate the deposition efficiency because the sample size was too small. Instead we had to use a value calculated from data by [46] (*Table S1*). The calculated value could have been influenced by the sample size used in [46] or other factors and therefore had associated uncertainty. Nevertheless the calculated value was not widely different from our findings for yearlings and older bears.

### 

#### Model validation

**Figure 4:**
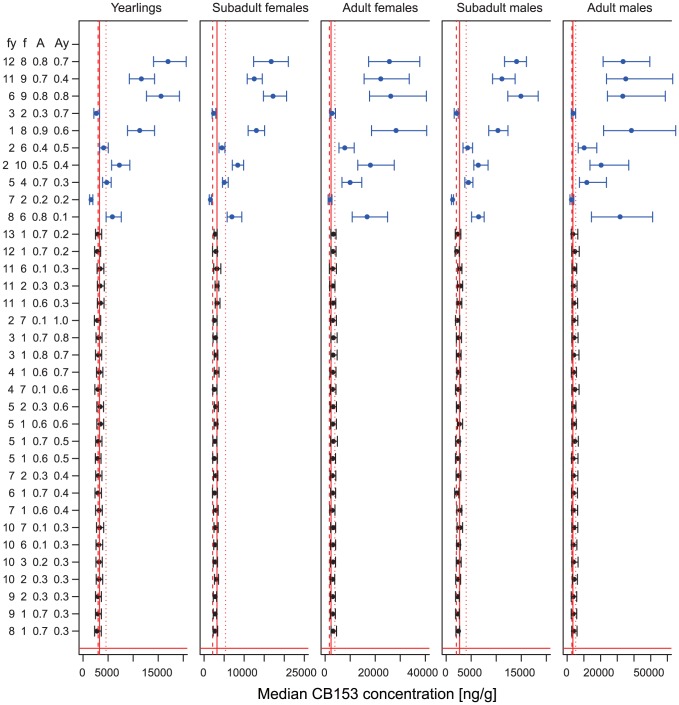
Medians of CB153 concentration of bear sub groups. Comparison between median observed CB153 concentrations in East Greenland polar bears (solid red lines) and CB153 concentration simulated using the best-fitting parameter combinations (black) or randomly selected parameter combinations (blue). Quartiles are shown as bars for model predictions and as dashed red lines for East Greenland bears.

The medians of CB153 concentration of each group of polar bears: yearlings, subadult females, subadult males, adult females and adult males matched fairly well with the East Greenland data when using the ANCOVA selected parameter sets (Table 1), but not for some of the other (randomly chosen) combinations (Fig. 4). The existence of parameter sets that produce not only matching time trend of pooled data but also of the sex and age dependent patterns supports the validity of the model structure and parameterization.

#### Sensitivity analysis

The sensitivity analysis revealed that the mean CB153 body burdens were indeed particularly sensitive to variations in the four focal parameters (*Figure S2*); the body burdens of yearlings were most sensitive to the field metabolic factor of yearlings, *f_Y_*, the deposition efficiency of yearlings *A_Y_* and further on by the energy contained in a gram of seal blubber (*E_s_*), and the weight of the yearlings (*W_Y_*). Similarly, the mean CB153 body burdens of older bears depended mostly on three parameters: the energy contained in a gram of seal blubber (*E_s_*), the field metabolic factor *f_A_* and the deposition efficiency, *A_A_*.

While these results were expected, the analysis pointed out the importance of the energy content of food. We have set the energy content of seal blubber to 37.8 kJ/g (or 9 kcal/g) based on the assumption that it consists of 100% blubber and is entirely digestible. We did not have sufficient data to include detailed feeding preferences (including various degree of contamination, energy content and digestibility of all types of food that East Greenland bears regularly feed on), but adding such details in future would help to improve the estimations of energy requirement.

Finally, the sensitivity analysis showed that the body burdens of yearlings were also strongly dependent on their weight, because their energy requirement is calculated on the basis of their body mass. The East Greenland bear data set did not contain data on body mass and therefore we based our calculations on a value obtained from the literature. This may have had an impact on the estimation of *f_Y_* as discussed above.

For our purpose of predicting the long-term time trend in CB153 adipose tissue concentrations across the population, it was necessary to couple the bioaccumulation model with population dynamics. At this stage we have not related the vital rates to the food supply of the bears and have instead assumed that bears forage according to their requirements. However it has been shown that longer ice free periods may result in decreasing litter sizes with direct effects on the population viability of polar bears [9]. Decreased survival in connection to prolonged fasting has also been demonstrated in male polar bears using a modelling platform Niche Mapper [65]. Our individual based model provides a platform that would allow for researching these issues in the context of a whole population in the future.

We aimed to construct a model of low complexity and a lowest possible number of parameters. By using only a single parameter to model the bioaccumulation - a parameter that is measurable in large mammals such as bears as Christensen et al. [36] demonstrated - we avoided the necessity of using a number of physiological and pharmacokinetic parameters derived from laboratory animal studies. Such an approach helped us to avoid the uncertainty associated with cross-species extrapolation. Using a single measurable parameter also increases the likelihood of such measurement being undertaken in relation to polar bears in future. In view of our having quantified the relationship between deposition efficiency and field metabolic rate this would then allow further refinement of our results regarding the field metabolic rate. However a physiologically based pharmacokinetic (PBPK) model has been developed for polar bears in the past [66] and using such a model would be advisable in order to understand the physiological processes that lead to the relatively low deposition of the highly persistent congener CB153 into polar bear adipose tissue.

Using a contaminant as a chemical marker of food intake has proven to be feasible. While we used CB153 in polar bears, we believe that our method, if adapted, could be used in other species using similar or other suitable contaminants. Although our model is based on many assumptions which carry varying degrees of certainty, we have pointed out an alternative, non-invasive way of estimating the energy requirements using contaminant monitoring data in relation to a species that is otherwise very difficult to study in the field.

In conclusion, the field metabolic rate of older East Greenland polar bears may be as low as 2^x^BMR under the assumption that polar bears deposit around 30% of the consumed CB153 into their adipose tissue. However, if they deposited only 10% of consumed CB153 into their adipose tissue their indicated field metabolic rate would be three times higher than previously assumed. This finding is of concern especially in view of the on-going climate changes, where polar bears will have less access to feeding opportunities. Polar bear yearlings could be in particular vulnerable to starvation and reduced food intake since their per unit body weight energy needs appear to be even higher than the energy needs of older bears, possibly reaching 11^x^ BMR. While our model is too simplistic to provide precise estimates we would like to point out the necessity of further research into this area. It is essential to know how much food polar bears consume in order to assess the impact of potential loss of foraging opportunities. A direct measurement of metabolic rates using the doubly tritium labelled water method at different seasons and for different age groups and sexes would be extremely valuable. Likewise, researching activity budgets together with data from GPS equipped polar bears on movements (which were disregarded in our model) coupled with spatial modelling or using the method by [65] for all seasons of the year could provide further insight into the amount of energy polar bears spend when roaming their natural habitat.

## Supporting Information

Figure S1 CB153 concentration in blubber of East Greenland seals. Black dots: field measurements, lines: linear model predictions for various age classes of seals.
(EPS)

Figure S2 Sensitivity analysis. Change (%) in mean CB 153 body burdens in yearlings and older bears following the change of parameters by +10% (red plus symbol) and - 10% (black minus symbol) of the reference value. Dashed red lines indicate standard deviation (in%) in the body burdens in the population when using original parameter values.
(EPS)

Table S1 Parameter values and references.
(DOC)

File S1 Model code – text file.
(TXT)

File S2 Model code – NetLogo file.
(NLOGO)

Text S1 Ringed seal contamination & growth.
(DOC)

Text S2 Overview, Design & Details (ODD) protocol.
(DOC)
