# Supplementary material for: Field Metabolic Rate and PCB Adipose Tissue Deposition Efficiency in East Greenland Polar Bears Derived from Contaminant Monitoring Data
Source: PLoS One. 2014 Aug 7;9(8):e104037. doi: 10.1371/journal.pone.0104037 (PMC4125222; doi:10.1371/journal.pone.0104037)
Supplement: Table S1 — Parameter values and references. (DOC) [file pone.0104037.s003.doc]

**Table S1. Parameter values and references.**

| **Parameter** | **Value** | **Units** | **Parameter meaning** | **References & notes** |
| --- | --- | --- | --- | --- |
| *σ_y_* | 0.916 | - | survival probability of a yearling | [43] |
| *σ_c_* | 0.57 | - | survival probability of a cub | assumption - cub survival increased |
| *β_0_* | 0.437 | - | breeding probability of female with status 0 | [43] |
| *β_12_* | 0.104 | - | breeding probability of female with status 1 or 2 | [43] |
| *f* | 1-10 | - | field metabolic energy requirement factor | estimated via calibration |
| *fy* | 1-13 | - | field metabolic energy requirement factor for yearlings | estimated via calibration |
| *m_cc_* | 469 | g/day | daily consumption of milk by cub | [64], [69] ref. in Supporting Information S2 |
| *m_cy_* | 131 | g/day | daily consumption of milk by yearling | [64], [69] ref. in Supporting Information S2 |
| *E_mc_* | 16.9 | kJ/g | energy content in milk for yearling | [64], [69] ref. in Supporting Information S2 |
| *E_my_* | 12 | kJ/g | energy content in milk for cub | [64], [69] ref. in Supporting Information S2 |
| *W_sm_* | 54.5 | kg | asymptotic weight | Data (seals in E Greenland 1986-2009) |
| *a_sm_* | 0.04247 | - | mass growth rate constant (seal male) | Data (seals in E Greenland 1986-2009) |
| *b_sm_* | 0.16599 | - | fitting constant (seal male) | Data (seals in E Greenland 1986-2009) |
| *W_sf_* | 48.1 | kg | asymptotic weight of ( seal female) | Data (seals in E Greenland 1986-2009) |
| *a_sf_* | 0.1114 | - | mass growth rate constant ( seal female) | Data (seals in E Greenland 1986-2009) |
| *b_sf_* | 0.2583 | - | fitting constant (seal female) | Data (seals in E Greenland 1986-2009) |
| *a_Tbl_* | 0.365 | - | regression parameters for seal blubber calculation | Data (seals in E Greenland 1986-2009) |
| *b_Tbl_* | 1.564 | - | regression parameters for seal blubber calculation | Data (seals in E Greenland 1986-2009) |
| *E_s_* | 37.8 *(9)* | kJ/g *(kcal/g)* | energy content of seal blubber | [10] |
| *F_milkC_* | 0.275 | - | proportion of fat in milk in cub | [69] ref. in Supporting Information Text S2 |
| *F_milkY_* | 0.206 | - | proportion of fat in milk in yearling | [69] ref. in Supporting Information Text S2 |
| *Ρ* | 0.67 | - | ratio of contaminant in fem. adipose tissue to milk | [46] |
| *A_c_* | 0.23 | - | proportion of CB153 deposited into adipose tissue (cub) | [46] |
| *A_y_* | 0.1-1 | - | proportion of CB153 deposited into adipose tissue (yearling) | estimated via calibration |
| *A* | 0.1-1 | - | proportion of contaminant deposited into adipose tissue | estimated via calibration |
| *μ_sm_* | 0.13 | - | mortality of adult male bear | [43] |
| *μ_m_* | 0.067 | - | mortality of subadult male bear | [43] |
| *μ_sf_* | 0.084 | - | mortality of adult female bear | [43] |
| *μ_f_* | 0.053 | - | mortality of subadult female bear | [43] |
| *π_1cub_* | 0.276 | - | probability of single cub in a litter | [70] ref. in Supporting Information Text S2 |
| *F_f_* | 0.28 | - | fat proportion in a female bear | assumption this study |
| *F_pf_* | 0.4 | - | fat proportion in a pregnant female bear | [46] |
| *F_fc_* | 0.23 | - | fat proportion in a female bear with cubs | assumption this study |
| *F_fy_* | 0.28 | - | fat proportion in a female bear with yearling | assumption this study |
| *F_m_* | 0.28 | - | fat proportion in a male | [71] ref. in Supporting Information Text S2 |
| *W_pf_* | 371 | kg | weight of a pregnant female | [46] |
| *W_fc_* | 161 | kg | weight of a female with cubs | [46] |
| *W_bm_* | 389 | kg | asymptotic weight of bear male | [44] |
| *k_bm_* | 0.303 | - | mass growth rate constant | [44] |
| *A_bm_* | -1.245 | - | fitting constant | [44] |
| *W_bf_* | 185 | kg | asymptotic weight of bear female | [44] |
| *k_bf_* | 0.58 | - | mass growth rate constant | [44] |
| *A_bf_* | -0.578 | - | fitting constant | [44] |
| *F_c_* | 0.11 | - | fat proportion in a cub | [46] |
| *F_y_* | 0.28 | - | fat proportion in a yearling | [46] |
| *W_c_* | 43.5 | kg | weight of a cub | [46] |
| *W_y_* | 90 | kg | weight of a yearling | [46] |
| *h_as_* | 18 | % | % of seals, 3-19yrs, among all seals killed by a bear/year | [20] |
| *h_sp_* | 56 | % | % of seal pups among all seals killed by a bear/ year | [20] |
