# Supplementary material for: Field Metabolic Rate and PCB Adipose Tissue Deposition Efficiency in East Greenland Polar Bears Derived from Contaminant Monitoring Data
Source: PLoS One. 2014 Aug 7;9(8):e104037. doi: 10.1371/journal.pone.0104037 (PMC4125222; doi:10.1371/journal.pone.0104037)
Supplement: Text S1 — Ringed seal contamination & growth. (DOC) [file pone.0104037.s006.doc]

**Supporting Information Text S1. Ringed seal contamination & growth.**

## **Predicting the age- and time-dependent concentrations of CB 153 in ringed seals.** We have started the analysis with model that included the independent variables year, age class and sex and all their interactions. This full model was simplified by removing the non-significant terms, starting with the interactions. Finally we obtained a simple model, log_10_ [CB 153] ~ year × age class (F _12, 232_ = 7.518, p<0.0001. R^2^=0.28). This model was used to predict the concentration of CB 153 in blubber for every age class of seal and each year in the period 1986–2010. The highest concentrations were predicted for the oldest age groups (Figure S1).

***Growth parameters of East Greenland ringed seals.*** To relate the seals’ age to their weight and to make it possible to calculate the overall amount of CB 153 contained in blubber of each seal, we determined sex dependent growth parameters of the sampled seals by fitting the age and weight of the seals with a growth curve used for seals in Svalbard [38, 39]:

$W_{seal}=W_{s}(1-e^{-a_{s}(age-x_{0})})^{b_{s}}$ [S1]

where *W*_seal_ is the weight of a seal [kg], *W*_s_ the asymptotic weight, *b*_s_ the fitting constant [years]; *x*_0_ was estimated to -0.61and represents prenatal growth [38]. Blubber content of the seals in the data was subsequently estimated using a relationship developed for ringed seals [67]:

$bl=a_{bl}\sqrt{\frac{L}{W}}d+b_{bl}$ [S2]

*bl* is blubber [%], *L* is seal length [cm], *W* is seal weight [kg], and *d* blubber depth [cm]; *a*_bl_ and *b*_bl_ are regression parameters estimated for ringed seals as 5120 and 8.53, respectively [38]. Because we are not using length and blubber depth in the model (in order to keep the number of parameters low) we fitted the estimations of blubber percentage in seals calculated with the relationship above with another linear regression to obtain the total amount of the seal blubber, *T*_bl_ [kg], dependent on weight of the seals only:

$T_{bl}=a_{Tbl}W+b_{Tbl}$ [S3]

where *a*_TB_ and *b*_TB_ are regression coefficients. The resulting values of these parameters are presented in Table S1. The seal blubber of the East Greenland seals contained on average 93.5% lipids.

**References**

67. Ryg M, Smith TG, Oritsland NA (1990) Seasonal changes in body mass and body composition of ringed seals (*Phoca hispida*) on Svalbard. Canadian Journal of Zoology 68: 470-475
