# Supplementary material for: Field Metabolic Rate and PCB Adipose Tissue Deposition Efficiency in East Greenland Polar Bears Derived from Contaminant Monitoring Data
Source: PLoS One. 2014 Aug 7;9(8):e104037. doi: 10.1371/journal.pone.0104037 (PMC4125222; doi:10.1371/journal.pone.0104037)
Supplement: Text S2 — Overview, Design & Details (ODD) protocol. (DOC) [file pone.0104037.s007.doc]

**Supporting Information Text S2. Overview, Design & Details (ODD) protocol.**

***1.1 Model description***

This model description follows a standardized protocol for describing individual and agent-based models - the ODD (Overview, Design concepts, Details) protocol [40, 41].

*Purpose.* The purpose was to produce predictions of CB153 concentrations in adipose tissue of a whole population of polar bears in East Greenland between 1986 and 2009.

*Entities, state variables and scales.* The only entities in the model were polar bear individuals, two years old or older. Bears were characterized by their *age* (2–30 years), *sex*, yearly requirement of *energy* (equal to *E_FC_* for females with cubs, *E_FY_* for females with yearlings and *E_A_* for all other bears), weight *W* [kg], pregnancy status *“p”* (true or false), the proportion of storage blubber “*fat”*, and total body burden of CB153, *B_B_* [ng]. The reproductive status “*status”* was set to zero for female with no offspring, to one for female with cubs, two for females with yearlings and five for males.

Cubs and yearlings were not represented as individual agents because their contamination level was dependent on the contamination level of their mother. Instead, the following variables of females represented the attributes of their offspring: number of offspring,” *ofno”* [0, 1 or 2], body burden of a cub, *B_C_*, body burden of a yearling, *B_Y_* , energy needed by a yearling, *E_y_*, and the number of days of survival for each offspring (“*days_1_”* and “*days_2_”*). Siblings (the offspring born in the same litter) were assumed to have equal milk consumption rate, energy requirement and weight and thus also their contaminant body burden equaled. The model did not incorporate any spatial aspects and proceeded in annual time steps. We simulated the period between 1986 and 2009. The only environmental variable was the content of CB153 in seals.

## **1.2 Process overview and scheduling**

The model consisted of submodels as described below. These were processed during each time step (year) in the order as presented in the text. In all submodels the order of processing the single agents (bears) was randomized and variables were updated immediately. For the implementation of the submodels and references, see section 1.6 below.

***Offspring survival and pregnancy***. First, it was determined whether the polar bear cubs would survive the simulated year and if not, for how many days of the year they survived. Second, it was determined whether females that were not pregnant would become pregnant during the simulated year.

***Update weight*** and ***Update blubber****.* In *Update weight* the weight of bears was calculated according to a von Bertalanffy growth function or set according to reproduction status. *Update blubber* served for updating the proportion of body fat of each bear according to sex and status.

***Set energy requirements***. This procedure calculated the energy requirements of each bear according to its reproductive status, weight and, in female bears, for how many days they lactated.

***Feed*.** Each bear satisfied its annual energy requirements by feeding on seals. With each seal consumed, the bears correspondingly increased their contamination burden by the amount present in the seal blubber multiplied by deposition efficiency. All bears satisfied their energy needs, i.e. there was no competition for resources incorporated in the model.

***Lactation transfer***. The proportion of contaminants that was transferred to the milk was subtracted from the total body burden of lactating females. The subtracted amount depended on the number of offspring, their age and the number of days they survived. The contamination in the offspring was also updated.

***Population dynamics.*** Bears survived or died according to given survival probabilities, irrespective of energy status or contaminant load. Females with surviving yearlings weaned them. For each weaned yearling a new two years old individual was created (sex assigned randomly). The mating status and pregnancy status of females were updated.

***Update age***.

***Update weight*** and ***Update blubber*** (again)*.* (See description above).

## **1.3 Design concepts**

*Basic principles.* The basic principles implemented in our model were feeding of polar bears on seal blubber, followed by partial deposition of the CB153 contained in consumed blubber and the subsequent transfer of contaminant from mothers to offspring via lactation.

*Emergence.* The contamination levels in individual adult polar bears emerged from the interaction of their feeding behavior and their reproductive activity.

*Stochasticity.* To reflect environmental variation, demographic rates were based on data collected in the period 2001–2006 from the Southern Beaufort Sea polar bear population [43] (with the exception of cub survival probability, see section 1.6 Submodels for details). The exact date of offspring death was a randomized variable and affected the female contamination level. The sex of the seals predated by each polar bear was chosen randomly.

*Observation.* The concentration of CB153 was recorded yearly for individual bears. We only used the output for the period 1986–2009 as our model results.

The modeled polar bears had no adaptive behavior, thus the design concepts *adaptation*, *prediction*, *sensing*, and *learning* as proposed in [40, 41] did not apply. We also neglected any possible interactions (such as competition, territoriality etc.) among bears with the exception of the mother – offspring contaminant transfer and therefore the concepts *interactions* and *collectives* as in [40, 41] did not apply either.

## **1.4 Initialization**

The bear population size in East Greenland is unknown, although it has been roughly estimated at 2,000 individuals [68]. We used this value as the initial population size. The starting year was 1950 and simulations were run for 60 years. The early start date for simulations was necessary to reach a stable age structure in the population and also to allow for the accumulation of contamination in polar bears to the 1986 level.

The sex and age (2-20 years) of the bears were assigned randomly. Initial status of adult females was set randomly as well as the number of their offspring. Weight was calculated according to von Bertalanffy equation for polar bears [44] and the fat proportion was determined using the *Update blubber* submodel (see section 1.6). The initial contaminant concentration of all bears was set to 0.

## **1.5 Input data**

Contamination of modeled polar bears was driven by seal consumption. The contamination of each seal age class in every year of simulation was predicted by the linear model fitted to the available data on ringed seal contamination as described in Supporting Information Text S1.

***1.6 Submodels***

For parameter symbols, definitions, default values, and sources for parameterization, see Table S1.

***Offspring survival and pregnancy****.*  The survival of the offspring and the possibility of pregnancy in females without offspring were determined according to probabilities as in Table S1 (values mostly from [43]). Whether offspring would survive the simulated year and which females would become pregnant was determined in order to allow accurate subsequent calculation of the energy demands of females (we accounted for access energy needed during pregnancy and for production of maternal milk). The exact number of days of offspring survival was equal to 365 (for a surviving offspring). Because the data by [43] were not seasonal we assumed a uniform probability of death during the whole year. Thus if offspring was predicted to die during the modeled year the number of days of its survival was randomly chosen from the interval [1-365]. Whether the offspring survived and the exact number of survival days was determined independently for each sibling. Single adult females and females who would lose the offspring during the simulated year could become pregnant according to probability *β_0_* and *β_12_* , respectively (Table S1). Neither survival nor breeding was connected to energy levels or contamination. It should also be noted that the survival probability of cubs *σ_c_* had to be increased to 0.57, compared to that in [43] in order to establish a slowly growing population.

**Set energy requirement**. The model did not include individual energy budgets, since corresponding data were not available. Annual energy requirement was only included as a state variable to determine the amount of seal blubber a bear consumed within a year, which in turn determined its contamination. The yearly energy requirement of an adult bear, *E_A_* [kJ], was calculated based on the Kleiber’s rule [45] as:

$E_{A}=365 \kappa f_{A} 70 W^{0.75}$ [S4]

where *W* is the mass [kg] of the bear, κ converts kilocalories to kilojoules and equals 4.2 and *f_A_* is a parameter adjusting for the field metabolic rate, further on referred to as “field metabolic factor” in bears two years old and older. The value of *f_A_* is not very well known for East Greenland polar bears and therefore we tested the influence of its value on the output.

In order to reflect higher energy needs of reproducing females, the yearly energy demand of females with cubs, *E_FC_* [kJ], or with yearlings, *E_FY_* [kJ], was equal to *E_A_* plus the total amount of energy contained in the milk produced by the female during the year (we neglected any additional energy expended on taking care of offspring because of lack of data):

$E_{FC}=E_{A}+m_{cc} E_{mc}\text{ }\sum_{\text{i}=1}^{2} \text{days}_{\text{i}}$ [S5]

$E_{FY}=E_{A}+m_{cy} E_{my}\text{ }\sum_{\text{i}=1}^{2} \text{days}_{\text{i}}$ [S6]

where *days_i_* is the number of days each offspring survived and *i* the number of the first and second offspring,  *E_mc_* (*E_my_*) is the energy content of milk for a cub (yearling) per gram [kJ/g], and *m_cc_* (*m_cy_*) the consumption of milk by a cub (yearling) [g/day] [64, 69] (Table S1). Because the energy demand of a growing cub was otherwise difficult to determine we assumed that in the first year of life the daily energy need equaled the energy gained from daily milk consumption as published by [64, 69]. Thus the contamination in the bodies of cubs originated only from milk. Because yearlings feed on seals and are also breast fed it was necessary to estimate the burden of CB153 originating from both these sources. The amount of energy gained by seal consumption (*E_YS_* ) [kJ/year] during a year was calculated as the difference between the yearly energy requirement calculated in eq.(S4) (using age specific parameters for yearlings) and the energy received from nursing as published in [64, 69]:

$E_{YS}=365 \left(\kappa f_{Y} 70 W_{Y}^{0.75}-m_{cy} E_{my}\right)$ [S7]

The ratio between the yearling’s and mother’s annual consumption of seals, *ε*, was thus calculated:

$\varepsilon=\frac{E_{YS}}{E_{FY}}$ [S8]

According to this ratio, combined with the contribution from milk, the yearling was subsequently assigned its PCB contamination level in the *Lactation transfer* submodel (eq. S16).

**Feed.** The yearly energy requirement (*E_A_, E_FC_* or *E_FY_*) was satisfied by consuming ringed seals. To simplify matters we assumed that only the blubber of the seal is consumed, as this is often the case in nature [2]. Each bear in the model would enter a cycle of catching seals whose sex was randomly determined and whose age was determined according to the hunting preferences of polar bears. We used information on the percentage of seals of different age categories among all seals in the typical annual kill of a polar bear [20] to model the hunting preference of bears. The categories were: seal pups (0 years), 1 – 2 years old and 3 - 19 years old. The exact age of the seals within these categories was random. The concentration of CB153 in blubber that corresponded to the age of the seal and the given year, *C_Seal_* [ng/g], was taken from a list of concentrations predicted from data as described in SI Text S1 (values included in the program code presented in Supplementary Information Files S1 and S2). The total amount of contaminant in the blubber of each seal, *I_C_* [ng], depended on the concentration of PCB in the blubber, *C_Seal_* [ng/g], and the total weight of its blubber *T_bl_* [kg] (which has been calculated according to the age of the seal using equations (S1) and (S3)):

$I_{C}=10^{3} T_{bl} C_{Seal}$ [S9]

For years 1950–1985 we used the 1986 contamination concentrations in ringed seals from Ittoqqortoormiit (Scoresby Sound), because no data was available on the contamination of seals in East Greenland prior to 1986. As a simplification we assumed that the seal blubber consisted of 100 % lipids (the mean lipid content in blubber of ringed seals in samples from East Greenland was close to 100%, as noted in Supplementary Information Text S1). The energy gain G [kJ] from each seal was equal to:

$G=10^{3} T_{bl} E_{s}$ [S10]

where *E_s_* was energy per gram of seal blubber [kJ/gram].

The bear repeatedly consumed seals until it satisfied its annual energy demand. Seals were not assumed to be a limited resource and competition for them did not occur in the model. The amount of CB153 that was finally incorporated in the adipose tissue of a polar bear depended on the value of *A_A_*, the deposition efficiency. The values of deposition efficiency *A_A_* tested were [0.1, 0.2 – 1], which corresponded to [10, 20 – 100] % of contaminant being stored in the adipose tissue. For cubs, the deposition efficiency was set to 0.23, according to our calculations based on literature findings [46]. In yearlings we tested various values of deposition efficiency *A_Y_* [0.1, 0.2 – 1].

We did not model any other explicit elimination pathway.

**Lactation transfer**. The body burdens of females with offspring decreased during the lactation period*.* The offspring on the other hand consumed an equivalent amount of contaminant from the mother and accumulated it partially in their adipose tissue. We assumed that the amount of the transferred contaminant, *M*, was dependent on the concentration of CB153 in the blubber of females. However because the time step of our simulation is one year and the mothers of cubs undergo a massive change in weight and fatness during that period, we used an average weight calculated from the weight of pregnant females *W_pf_* and weight of females with cubs *W_fc_* (Table S1) and also averaged the fat content of the two (*F_fc_* in female with cubs and *F_pf_* in pregnant female, Table S1) to calculate the average concentration of CB153 in the adipose tissue of a female with cubs:

$C_{fc}=\frac{B_{B}}{10{}^{3} (\frac{W_{fc}+W_{pf}}{2})(\frac{F_{fc}+F_{pf}}{2})}$ [S11]

Subsequently the body burdens *B_B_* of the mother decreases by the amount of contaminants transferred via the milk, *M_c_* [ng], during the year:

$M_{c}=C_{fc} m_{cc} F_{milkc} \rho\sum_{\text{i}=1}^{2} \text{days}_{\text{i}}$ [S12]

where *m_cc_* is the daily consumption of milk by a cub [g/day] and *F_milkc_* is the proportion of fat in milk for the cubs (Table S1). Parameter ρ was the ratio of contaminant concentrations in milk fat/ female adipose tissue as calculated using data published in [46] for sum PCBs.

Contamination transfer *M*_y_ for females with yearlings, was determined accordingly. Female weight *W* was calculated using equation (S17) described below (submodel *Update weight*) and the contamination concentration *C_fy_* [ng/g] for a female with yearlings was calculated as follows:

$C_{fy}=\frac{B_{B}}{10^{3}WF_{fy}}$ [S13]

The CB153 body burdens of a female with offspring were decreased by this amount (*M_y_*,[ng]) of transferred contaminant in milk during a year:

$M_{y}=C_{fy} m_{cy} F_{milky} \rho\sum_{\text{i}=1}^{2} \text{days}_{\text{i}}$ [S14]

where *m_cy_* was the daily consumption of milk by a yearling [g/day] and *F_milky_* was the proportion of fat in milk for a yearling (Table S1).

The body burden of cubs *B_C_* [ng] was determined for cubs that survived the simulated year as:

$B_{C}=365 C_{fc} m_{cc} F_{milkc} \rho A_{c}$ [S15]

where *A_c_* was the deposition efficiency of CB153 into the adipose tissue of a cub.

Because yearlings received contamination through both milk and seal blubber, their body burden *B_Y_* [ng] was calculated as:

$B_{Y}=A_{y}(365 C_{fy} m_{cy} F_{milky} \rho+\varepsilon\sum_{1}^{n} (I_{C})_{n} )$ [S16]

where *A_y_* is the deposition efficiency of CB153 into the adipose tissue of a yearling and *ε* was the ratio of energy covered by seal consumption in a yearling to yearly energy requirement of its mother; *I_c_* is the amount of CB153 in each seal hunted by the mother and *n* the number of seals consumed by the mother and its yearling.

**Population dynamics.** In the first step the bears that were to die during the simulated year were selected according to individual death probabilities (Table S1)*.* The maximum age was 30 years. In the next step females with a surviving yearling or two weaned them and changed status to “without offspring”. For each weaned offspring a new two years old individual with corresponding body burdens was created. Females that weaned offspring would not become pregnant during the same year because in nature they would be accompanied by offspring for about half a year longer. We assumed that the energy and contamination contribution from breast feeding were negligible during the third year of life and thus the cubs were modeled as independent agents from two years and onwards. Finally, the status of other females was updated: females with surviving cubs changed status to “with yearlings” and pregnant females changed status to “with cubs” (they were assigned either one cub with the probability π_1cub_ or two cubs with probability 1- π_1cub_ ( [70], Table S1) .

**Update age**. The age of all bears was incremented.

***Update blubber****.* The proportion of polar bear fat was updated according to the values mostly from [46, 71] as in Table S1 .

***Update weight****.* This procedure calculated weight according to von Bertalanffy growth function used for polar bears previously [44]:

$W=W_{bi}(1-e^{-k_{bi}(age-A_{bi})})^{3}$ [S17]

where *W* is the mass [kg] at age *age, W_bi_* is the asymptotic weight, *k_bi_* is the mass growth rate constant, *A_bi_* is the fitting constant (years) and *i* equaled *f* (females) or *m* (males) (Table S1). The weight of pregnant females and females with cubs was also adjusted. Because no information was available to us on the relationship between age and pregnancy weight and age and weight after nursing we used two average values *W_fc_* and *W_pf_* [46] (Table S1).

**File output**. For a random sample of 10% of the population the following variables were recorded to output files every simulated year: age, sex, body burden and concentration of CB153 in adipose tissue [ng/g].

**References**

68. IUCN/SSC Polar Bear Specialist Group (2002) Status of the polar bear. Pp. 21–35 In Luann, N.J., Schlep, S. and Born, E.W. (eds.). 2002. *Polar Bears: Proceedings of the 13^th^ Working Meeting of the IUCN/SCC Polar Bear Specialist Group, 23–28 June 2001, Nuuk, Greenland*. IUCN, Gland, Switzerland and Cambridge, UK.

69. Derocher AE, Dennis Andriashek D., Arnould JPY (1993) Aspects of milk composition and lactation in polar bears. Canadian Journal of Zoology 71: 561-567.

70. Hunter CM, Caswell H, Runge M, Regehr EV, Amstrup SC, et al. (2010) Climate change threatens polar bear populations: a stochastic demographic analysis. Ecology, 91(10), 2010, pp. 2883–2897

71. Polischuk SC, Norstrom, RJ, Ramsay, MA (2002) Body burdens and tissue concentrations of organochlorines in polar bears (Ursus maritimus) vary during seasonal fasts. Environmental Pollution 118 (1): 29-39.
